# Development of a Robot-Assisted TMS Localization System Using Dual Capacitive Sensors for Coil Tilt Detection

**DOI:** 10.3390/s26020693

**Published:** 2026-01-20

**Authors:** Czaryn Diane Salazar Ompico, Julius Noel Banayo, Yamato Mashio, Masato Odagaki, Yutaka Kikuchi, Armyn Chang Sy, Hirofumi Kurosaki

**Affiliations:** 1 Systems and Bioengineering Department, Faculty of Engineering, Maebashi Institute of Technology, Maebashi 371-0816, Gunma, Japan; czarynompico@gmail.com (C.D.S.O.); yamax.gmax@gmail.com (Y.M.); 2 Evelyn D. Ang-Institute of Biomedical Engineering and Health Technologies, De La Salle University, Manila 1004, Philippines; julius.banayo@dlsu.edu.ph (J.N.B.); armyn.sy@dlsu.edu.ph (A.C.S.); 3 Institute of Brain and Blood Vessels, Mihara Memorial Hospital, Isesaki 372-0006, Gunma, Japan; viconmx@gmail.com; 4 Manufacturing Engineering and Management Department, De La Salle University, Manila 1004, Philippines; 5 Gunma Industrial Technology Center, Maebashi 379-2147, Gunma, Japan; hirofumi.kurosaki@gmail.com

**Keywords:** Transcranial Magnetic Stimulation, robotic-assisted TMS, coil localization, 3D camera, capacitive sensors, tilt detection

## Abstract

**Highlights:**

**What are the main findings?**

- A robotic-assisted TMS system using a 3D camera to detect facial landmarks can successfully locate the C3 motor hotspot with minimal calibration.
- Sensor ratio balance and moderate contact pressure result in higher MEP amplitudes, indicating effective coil–scalp alignment and tilt detection.

**What are the implications of the main findings?**

- Low-cost textile capacitive sensors are able to provide real-time feedback on coil positioning and tilt, enabling safer and more reproducible TMS procedures.
- Robotic assistance has the potential to reduce setup time and operator dependency compared to traditional manual localization, improving efficiency and consistency in clinical and research settings.

**Abstract:**

Transcranial Magnetic Stimulation (TMS) is a non-invasive technique for neurological research and therapy, but its effectiveness depends on accurate and stable coil placement. Manual localization based on anatomical landmarks is time-consuming and operator-dependent, while state-of-the-art robotic and neuronavigation systems achieve high accuracy using optical tracking with head-mounted markers and infrared cameras, at the cost of increased system complexity and setup burden. This study presents a cost-effective, markerless robotic-assisted TMS system that combines a 3D depth camera and textile capacitive sensors to assist coil localization and contact control. Facial landmarks detected by the depth camera are used to estimate the motor cortex (C3) location without external tracking markers, while a dual textile-sensor suspension provides compliant “soft-landing” behavior, contact confirmation, and coil-tilt estimation. Experimental evaluation with five participants showed reliable C3 targeting with valid motor evoked potentials (MEPs) obtained in most trials after initial calibration, and tilt-verification experiments revealed that peak MEP amplitudes occurred near balanced sensor readings in 12 of 15 trials (80%). The system employs a collaborative robot designed in accordance with international human–robot interaction safety standards, including force-limited actuation and monitored stopping. These results suggest that the proposed approach can improve the accessibility, safety, and consistency of TMS procedures while avoiding the complexity of conventional optical tracking systems.

## 1. Introduction

Transcranial Magnetic Stimulation (TMS) is a widely used non-invasive brain stimulation technique for both research and clinical applications. TMS induces localized electric currents in cortical regions by generating rapidly changing magnetic fields, thereby modulating neuronal activity. It is commonly used for treating major depressive disorder (MDD), particularly for patients resistant to pharmacological therapy, and also clinically accepted in treating other neurological and psychiatric disorders, including postpartum depression (PPD) and substance use disorders (SUDs) [1]. High-frequency stimulation generally enhances cortical excitability, while low-frequency stimulation suppresses overactive areas. Several studies have reported a decrease in depressive symptoms following stimulation of regions such as the dorsolateral and medial prefrontal cortex [2,3].

Despite these advances, TMS efficiency and reproducibility have certain limitations. Even subtle deviations can reduce stimulation efficacy. Hence, accurate and stable coil placement is critical. Manual positioning can lead to operator fatigue, small drifts during long sessions, and inter-session variability, all of which compromise treatment consistency [4,5,6].

To address these challenges, neuronavigation and robotic systems have been developed. Advanced neuronavigation solutions often integrate MRI or fMRI data to guide coil placement with millimeter-level precision [7]. Recent robotic systems developed for TMS incorporate real-time force feedback and imaging. Examples include robots with multi-axis force sensors that regulate contact pressure [8], and spherical robots with neuro-navigation that use visual tracking to maintain precise alignment [9]. However, these systems are often costly, requiring equipment such as laser trackers and head markers that can compromise patient comfort. This study does not aim to achieve MRI-level anatomical targeting of specific cortical sulci or gyri. It focuses on improving the repeatability, accessibility, and functional consistency of scalp-based TMS coil positioning using a markerless robotic approach.

The present study proposes a low-cost, markerless robotic-assisted TMS system that combines a 3D depth camera with textile capacitive sensors. The system automatically detects facial landmarks to localize the motor cortex (C3) and employs a soft, compliant suspension structure with dual textile sensors to ensure even coil contact and detect tilt. Unlike recent similar systems that depend on force sensors or external trackers, this method emphasizes affordability, accessibility, patient comfort, and ease of use. This work describes the design, implementation, and preliminary evaluation of the system, with the aim of enhancing coil placement accuracy and reducing operator workload in future TMS applications.

## 2. Materials and Methods

### 2.1. System Overview

**Figure 1:**
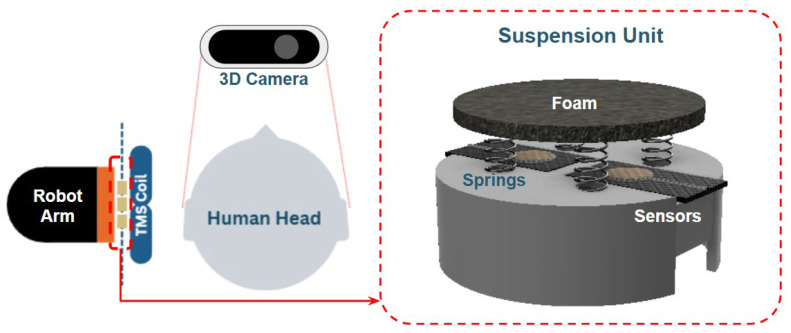
System overview showing camera and robot placement. Suspension platform housing the two capacitive sensors.

The proposed system integrates an igus ReBeL 5-DOF collaborative robot, Intel RealSense D435 depth camera, and two custom textile capacitive sensors placed on a suspension platform, which can be seen in Figure 1. The robot holds the TMS coil and adjusts orientation based on camera-derived anatomical landmarks and sensor feedback. The igus ReBeL collaborative robot provides repeatable and stable positioning as a commercially available platform compliant with international safety standards. Evaluation of intrinsic robot positioning accuracy and joint-level stability was beyond the scope of this work.

All system logic, data acquisition, and robot control were implemented in C# using Visual Studio 2022, with communication to the igus Robot Control (iRC) Release 13 software via the CRI (Container Runtime Interface) protocol. The RealSense SDK and OpenCV-Sharp handled image and depth processing, while DlibDotNet provided 68-point facial landmark detection.

### 2.2. Hardware Configuration

#### 2.2.1. igus ReBeL 5-DOF Cobot

An igus ReBeL (igus GmbH, Cologne, Germany) 5-DOF cobot serves as the primary actuator for holding and positioning the TMS coil. While 6-DOF robots offer full 3D orientation control, the ReBeL’s 5-DOF architecture limits independent pitch and yaw adjustments relative to the patient’s head. Accordingly, the control and sensing framework in this study was designed to maintain proper coil alignment and manage tilt within this constrained workspace.

As a collaborative robot, the ReBeL adheres to safety requirements outlined in ISO 10218-1:2011, ISO 10218-2:2011, and ISO/TS 15066:2016, which define functions such as monitored standstill, hand-guiding, speed-and-separation monitoring, and power-and-force limiting [10,11,12]. In compliance with these standards—particularly the ≤250 mm/s limit for safety-rated reduced speed—the robot was operated at only 10–20% of its maximum speed during all experiments. This ensured low-risk operation and stable, controlled coil handling.

**Figure 2:**
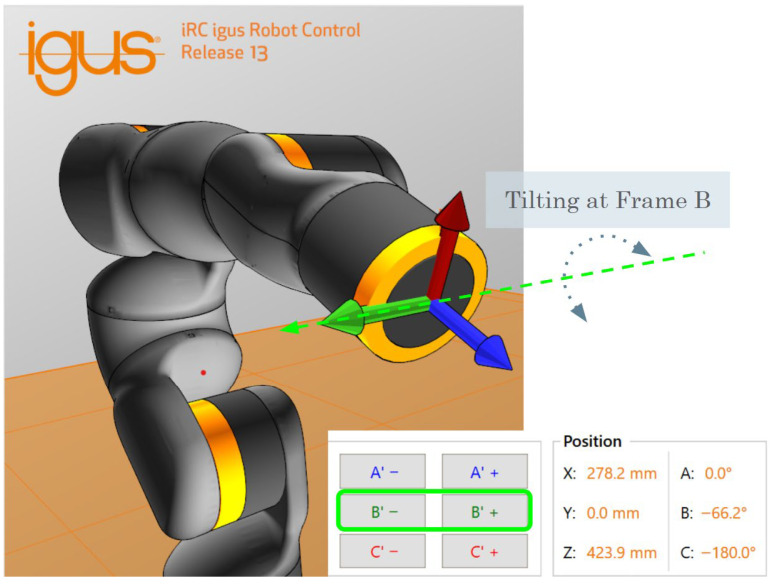
Visualization of the robot tool Cartesian, highlighting Frame B, taken from the iRC software.

For tilt manipulation, the end-effector orientation was controlled in Cartesian space using the robot’s tool coordinate system rather than by directly actuating a single joint such as A5, which is the fifth joint of the robot that changes the pitch. Specifically, the tool-frame orientation parameter B was adjusted to vary the coil tilt. This parameter B is displayed in Figure 2, corresponding to pitch rotation of the end effector in the robot’s Cartesian coordinate representation (X,Y,Z,A,B,C). Although geometric offsets accounting for the TMS coil thickness and the 3D-printed holder have not yet been incorporated into the tool model, preliminary calibration confirmed that tilt-dependent changes in motor-evoked potential (MEP) amplitude could still be reliably observed.

#### 2.2.2. Textile Capacitive Sensors

Textile capacitive sensors measure variations in capacitance resulting from deformation and changes in proximity between conductive layers. When integrated behind the TMS coil within a compliant suspension structure, these capacitance variations reflect changes in contact pressure and mechanical compression at the coil–scalp interface. Unlike rigid force or torque sensors, textile-based capacitive sensors provide a soft, distributed sensing surface that conforms to scalp curvature, thereby improving subject comfort and tolerance to minor positioning errors.

Compared to ultrasonic or infrared distance sensors, textile capacitive sensors do not require line-of-sight, are insensitive to hair coverage and surface reflectivity, and can be embedded directly into a compliant mechanical interface. These properties make them particularly suitable for close-contact human–robot interaction scenarios, such as TMS coil positioning, where continuous and stable scalp contact is required.

The textile capacitive sensors used in this study were custom-fabricated in-house by a former doctoral researcher in the same laboratory, in collaboration with the Gunma Industrial Technology Center. Each sensor consists of flexible conductive fabric layers separated by a compliant dielectric material, allowing measurable changes in capacitance in response to pressure or deformation. While the sensors employed in this system were custom-built, the design is readily replicable, and alternative fabric-based or capacitive sensors may be substituted, provided they offer stable analog outputs compatible with the data acquisition hardware. This flexibility supports the broader goal of maintaining affordability and adaptability across different research environments.

Two textile capacitive sensors were integrated between the robot flange and the TMS coil holder, with one sensor positioned at the upper and the other at the lower portion of the end effector. This dual-sensor configuration enables real-time estimation of coil tilt by comparing differential capacitance responses arising from asymmetric compression of the suspension structure.

Data processing for sensor reading:

- A 50-sample baseline for each sensor is collected for calibration
- Baseline is subtracted from real-time reading (capacitance count):

Adj_n_ = Raw_n_ − BaseLine_n_, (1)

- Normalized ratio between the two sensors is computed:

(2) $${Norm}_{n}=\frac{{Adj}_{n}}{{Adj}_{1}+{Adj}_{2}}$$

Tilt detection is based on asymmetric compression of the suspension structure. When the coil is approximately perpendicular (tangential) to the scalp surface, both textile sensors undergo similar deformation, resulting in comparable adjusted capacitance values. When the coil is tilted, one side of the suspension compresses more than the other, producing an imbalance between the upper and lower sensor readings. The normalized ratio therefore provides an indirect estimate of coil tilt relative to the local scalp surface.

With the total load (Adj_1_ + Adj_2_) normalized to unity, normalized values approaching 0.5 indicate balanced contact between the two sensors, corresponding to a coil orientation that is approximately tangential to the scalp. Deviations from this balance indicate increasing tilt.

An automatic tilt-correction algorithm utilizing this sensor ratio was implemented in the control software. However, this closed-loop correction was not activated during the experimental procedures reported in this study. The algorithm was functionally tested during system development to verify correct execution and safe behavior, but it was excluded from formal experiments to isolate and evaluate the sensing and estimation performance of the textile capacitive sensors themselves. Consequently, all tilt adjustments during experimentation were performed manually, and experimental validation of the automatic correction mechanism remains future work.

**Figure 3:**
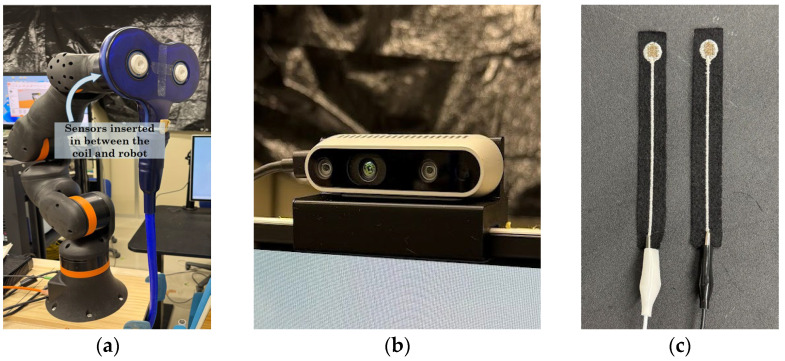
Hardware Components: (**a**) TMS coil attached to igus ReBeL 5-DOF cobot; (**b**) Intel RealSense D435 with 3D printed mount; (**c**) two custom textile capacitive sensors.

Overall, capacitive textile sensing was selected because it enables sensitive, continuous measurement of coil–scalp interactions without requiring rigid mechanical interfaces or point-force sensing. The combination of flexibility, durability under repeated compression, and spatially distributed sensing provides a richer representation of load distribution and coil orientation than single-point force sensors, supporting its suitability for robotic-assisted TMS applications. The overview of the hardware components are shown in Figure 3.

#### 2.2.3. Suspension System

**Figure 4:**
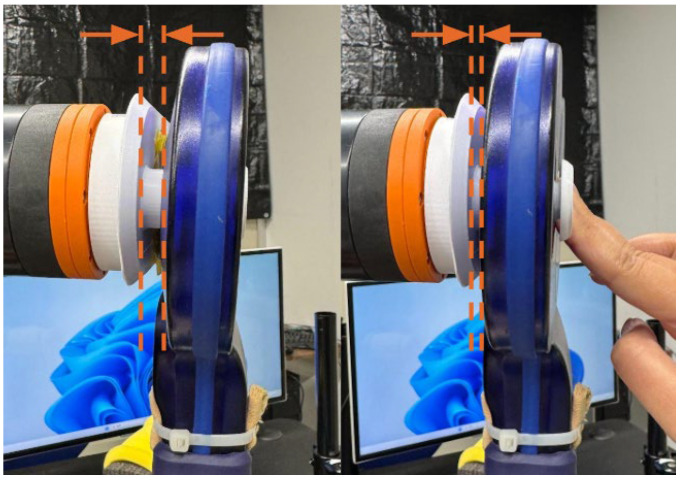
Subtle displacement of the TMS coil, visualized by the dotted lines, when pushed on the suspension system.

To ensure accurate load detection and consistent contact between the TMS coil and the scalp surface, a custom suspension system was designed and 3D-printed to support the textile capacitive sensors. Since the sensors were positioned behind the TMS coil, the direct change in capacitance upon coil–head contact was initially minimal, with the coil itself acting as a source of interference. Instead of filtering out this signal, the system design leveraged it as a form of load detection. Figure 4 displays the effect of the suspension system on the displacement of the coil when pushed.

The suspension structure consists of a lightweight 3D printed platform supported by soft polyurethane foam and compression springs. When the coil contacts the scalp, the mechanism compresses backward, pushing against the sensors and generating a capacitance change proportional to load. This “soft-landing” design also provides a cushioning effect, which improves safety and comfort during approach. This suspension system aids tilt estimation through differential readings across the two sensors.

### 2.3. Software Architecture and C3 Localization

The system was programmed in C# using Microsoft Visual Studio 2022. An Intel RealSense D435 (Intel Corporation, Santa Clara, CA, USA) [13] provided synchronized RGB–D data through the RealSense SDK 2.0 for real-time facial landmark detection. Although the D435 has no major technical advantage over other depth cameras, it is inexpensive, widely supported, and easily integrated with OpenCV and DlibDotNet. The system does not depend on this specific model and any depth camera capable of landmark detection would be suitable.

Facial landmarks were detected using the DlibDotNet library, a C# wrapper for the Dlib C++ machine learning library. This library provides pre-trained models for detecting key facial points [14], including the nasion and preauricular landmarks, which were then used to estimate the C3 position according to the international 10–20 EEG system. In this study, C3 was used as an anatomically defined reference location rather than a subject-specific functional motor hotspot. C3 was selected because it lies over the primary motor cortex in many individuals and is commonly used as an initial targeting point in both clinical and research TMS settings.

Functional motor mapping was not performed to redefine the optimal hotspot location. Instead, MEPs elicited during stimulation were used as a physiological confirmation that the coil was positioned over a motor-responsive cortical region, thereby validating the effectiveness and repeatability of the proposed localization approach. This strategy allowed evaluation of the system’s ability to reproducibly position the coil at a consistent anatomical target while avoiding confounds introduced by individualized functional mapping.

**Figure 5:**
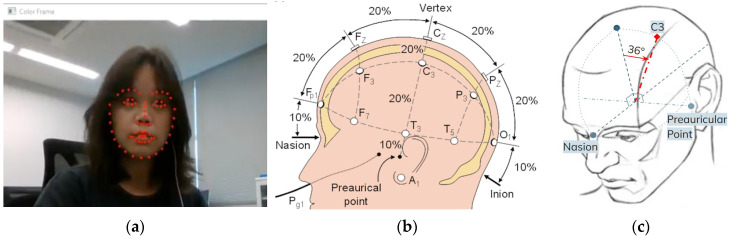
C3 Localization (**a**) Using DlibDotNet shape_predictor_68_face_landmarks.dat [14] to detect facial landmarks; (**b**) International 10–20 Method [15]; (**c**) Visualization of C3 estimation from 10–20 method.

Extracted facial landmarks for this project include the nasion, and right and left preauricular points along the jaw. Facial landmark detection and simplified 10–20 method are visualized in Figure 5.

#### 2.3.1. C3 Estimation Procedure

Because the inion cannot be captured in the camera view, the full 10–20 system was adapted using only visible landmarks. The procedure is:

1. Define the transverse plane.
  The distance between the left and right preauricular points is treated as 100% of the transverse arc (180° in the simplified angular model).
2. Approximate the vertex.
  Normally at 50% of the nasion–inion distance; here, it is estimated from the geometric midpoint of the preauricular points and the nasion, which also provides pitch/roll estimation.
3. Locate C3.
  In the 10–20 system, C3 lies 20% of the transverse distance from the vertex. In the simplified model, 20% of 180° is 36°. The C3 direction is therefore the 36° angle from the vertex toward the left ear.
4. Estimate head orientation.
  Using the nasion–ear triangle, pitch and roll are computed, enabling the C3 point to be projected onto a tilt-corrected anatomical plane.

Thus, C3 is estimated as the point lying 36° from the approximated vertex on the tilt-corrected transverse plane.

#### 2.3.2. Coordinate Computation

Because the 5-DOF cobot has limited translation along the depth (Z) axis, Z is treated as constant for the localization task. The target X–Y location is computed as:

Ear_x_ = RightEar_x_ − LeftEar_x_;     Ear_y_ = RightEar_y_ − LeftEar_y_ (3)

(4) $$\theta =Arctan\left(\frac{{Ear}_{y}}{{Ear}_{x}}\right)$$

(5) α=100cosθ

(6) $${Target}_{x}={MP}_{x}-\alpha tan(36+\theta )$$

(7) $${Target}_{y}={MP}_{y}+\alpha$$

**Figure 6:**
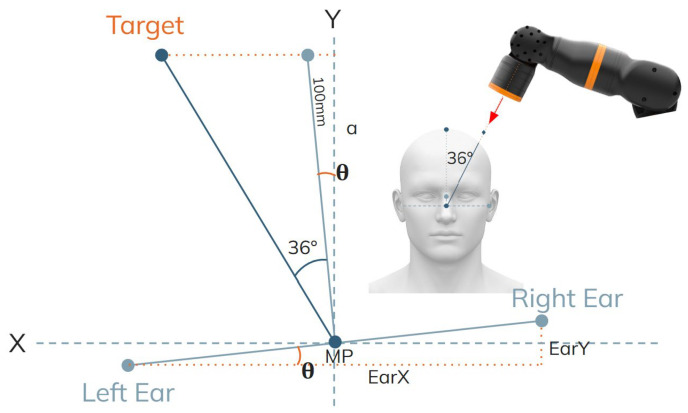
Visualization of C3 coordinate computation.

These equations can be visualized in Figure 6. The α is an allotted offset as the exact height of the head cannot be accurately detected. 100 mm from the midpoint of the preauricular points is the added offset so that the robot can simply move forward, toward the midpoint at the computed angle.

The resulting XYZ coordinates were sent via the iRC interface using the CRI protocol. Using a Renesas microcontroller board, textile sensors continuously collect capacitance counts to monitor contact and coil tilt. The control logic automatically stopped or retracted the robot whenever the sensor readings indicated excessive capacitance, ensuring safe interaction with the scalp.

### 2.4. Experimental Procedures

MEPs were recorded from the right first dorsal interosseous (FDI) muscle using surface EMG in a standard three-electrode bipolar configuration, with two electrodes placed over the muscle belly and a reference electrode on a nearby electrically neutral site. EMG signals were amplified (500× gain) and digitized at 5000 Hz using a laboratory-developed MATLAB R2024a-based acquisition system. Recordings were synchronized with TMS pulses via an external TTL trigger, with a 160 ms recording epoch per trial. Signals were preprocessed by voltage conversion (accounting for gain and 2.5 V DC offset), band-pass filtered (20–1000 Hz, 4th-order IIR), and notch filtered at 50 Hz (2nd-order IIR). Zero-phase digital filtering (filtfilt) was applied to avoid phase distortion.

MEP amplitude was defined as the peak-to-peak value (Vmax–Vmin) within a 20–40 ms post-stimulation window. Responses exceeding 50 μV were classified as valid MEPs. A MagStim 200 figure-eight TMS coil was mounted on the igus ReBeL cobot and positioned over the estimated C3 target based on camera-detected facial landmarks. Meanwhile, the textile sensors provided real-time feedback on coil–scalp contact.

#### 2.4.1. MEP Threshold Determination

To ensure comparable data across tilt verification testing, the resting motor threshold (RMT) for each subject was determined using standard clinical procedures. The coil was positioned at the C3 point, and ten single pulses, with resting periods, were delivered at a given intensity. If at least 5 of 10 pulses produced an MEP greater than 50 µV peak-to-peak, that intensity was defined as the RMT. If fewer or more responses were elicited, intensity was increased or decreased accordingly until a 50% response rate was achieved.

All subsequent stimulation was delivered at 120% of the participant’s RMT to ensure reliable suprathreshold activation while minimizing discomfort. For example, if the RMT determined is of 50% maximum stimulator output (MSO), 120% of the RMT was used in experimental setups, which in this case results in 60% MSO. It is important to note that different manufacturers and device models cause variations in stimulation output scale. The values reported in this study correspond specifically to the MagStim 200 system (Magstim Co., Ltd., Whitland, UK) [16], as it was the TMS model used for all experiments.

#### 2.4.2. C3 Localization Experiment

This experiment evaluated the accuracy and usability of the C3 localization program and was performed alongside the Tilt Verification Experiment. With the participant stabilized using a headrest, the program computed the XYZ coordinates of the C3 target, and the robot moved the coil to the calculated position. For this test, the final advancement to the scalp was performed manually until sensor-confirmed contact was reached.

After stimulation at a suprathreshold TMS intensity (120% RMT), MEP responses were recorded, whether present or absent. If no MEP was detected, the system was recalibrated and the procedure repeated. For each trial, the number of attempts, the number of camera recalibrations, and any relevant observations were documented.

#### 2.4.3. Tilt Verification Experiment

This experiment was conducted to verify the optimal coil tilt for effective stimulation, determine any other relationships between MEP responses and coil orientation, and assess the performance of the sensors in detecting tilt. Five healthy volunteers completed three trials each. For every trial, the robot positioned the coil at the C3 target coordinate.

Based on prior literature indicating that a ~45° orientation relative to the midsagittal plane optimizes corticospinal activation by aligning the induced field perpendicular to the central sulcus [17,18], the coil was first positioned at 45° and advanced until scalp contact was confirmed by the sensors. From this reference position, the coil was tilted superiorly and inferiorly in 1° increments, spanning ten total conditions (−4° to +5°).

Instead of simply changing the joint angles, tilt adjustments were applied by modifying the pitch orientation of the end-effector (Frame B), ensuring tilt changes occurred without altering the XYZ position of the end-effector. While coil thickness offset has not yet been fully integrated in this system, all tilt conditions represent consistent relative angular deviations from a uniform contact reference. Thus, while absolute orientations may include a small offset, comparisons across tilt conditions remain valid. Rest periods between trials reduced fatigue-related variability.

For every tilt angle variation:

- One suprathreshold TMS pulse was delivered;
- MEP amplitude and graph were recorded;
- Upper and lower textile sensor total load and normalized readings were logged.

## 3. Results

### 3.1. Motor Thresholds of Participants

Resting motor thresholds (RMTs) were successfully obtained for all five participants before the tilt verification experiment. All volunteers were male; four were in their early twenties and one in his late forties. RMT values ranged from 50% to 64% MSO, consistent with typical inter-individual differences in corticospinal excitability. These RMT values, which are listed in Table 1, were subsequently used to standardize the stimulation intensity for the tilt test at 120% RMT for each participant.

### 3.2. C3 Localization

**Figure 7:**
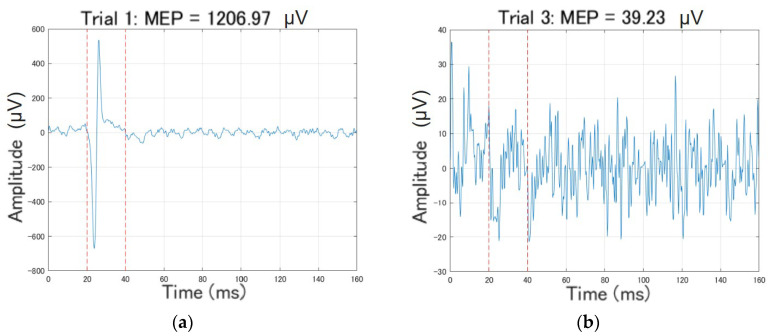
Contrasting MEP responses: (**a**) Good MEP response with clear peak and >50 µV amplitude; (**b**) Poor MEP response with no clear peaks, very noisy, and <50 µV amplitude. Dashed lines indicate the 20–40 ms post-stimulation window used for MEP evaluation.

All attempts to position the TMS coil using the developed C3 localization program were recorded, including the total number of trials and any notable deviations in coil placement or subject response. A stimulation attempt was considered successful when the MEP amplitude exceeded 50 µV, which is a commonly used physiological threshold for confirming activation of the affected muscles. Subjects reporting index-finger twitching were also taken into consideration when recording the MEP response as successful or not. MEP responses can be visually differentiated as shown in Figure 7.

Attempts and remarks for all subjects are summarized in Table 2.

### 3.3. Tilt Ratio Verification

During the tilt verification experiment, the program allowed pausing of sensor updates to record static values per trial. For each recorded angle, the following were documented: robot pitch angle, total load, normalized readings from each of the two sensors, and the resulting MEP amplitude.

Table 3 shows sample data from Subject A, Trial 3. The reference angle, which is the computed 36° from the subject’s vertex, is represented by a different value in the robot pitch angle. In this example, the reference angle in the robot pitch is 25°, which varies across trials due to head tilting.

**Figure 8:**
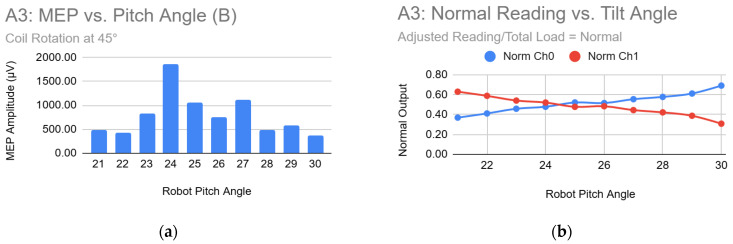
Subject A Trial 3: Graphs (**a**) MEP Amplitude vs. Robot Pitch Angle; (**b**) Normal Sensor Reading vs. Robot Pitch Angle.

Using the collected data, graphs were generated to illustrate the relationship between tilt angle, sensor values, and MEP amplitude, as shown in Figure 8. Complete plots for all subjects and trials are provided in Appendix A.

## 4. Discussion

This study evaluated the feasibility of a robotic-assisted TMS positioning system using textile capacitive sensors and a 3D camera to support coil alignment, contact monitoring, and tilt detection. The findings provide preliminary evidence that the system can (1) localize the C3 motor hotspot with minimal correction after initial calibration, (2) detect coil tilt through differential sensor ratios, and (3) identify a contact-pressure range (“total load”) associated with improved MEP responses. The implications of these results are discussed in detail below.

### 4.1. C3 Localization Reliability

Across all participants, the C3 localization program successfully positioned the TMS coil at the target location, requiring only minor calibration adjustments after initial setup. In most trials, a single positioning attempt was sufficient.

Subject A required additional attempts during the first trial due to an incorrect initial coil rotation instead of the recommended 45° for motor cortex stimulation, which prevented reliable MEP generation. Once corrected, localization accuracy was comparable to that of the other participants. Subjects B–E consistently achieved acceptable positioning with one attempt per trial after initial calibration, with Subject E representing the optimal user experience, requiring only one attempt in all trials.

This suggests that the computational method for determining C3 is generally reliable, and when combined with operator familiarity, its reproducibility improves across sessions. Minor calibration discrepancies are likely attributable to camera-related factors, such as depth noise or inaccuracies in point-to-pixel and pixel-to-point transformations. Future work should focus on improving data acquisition stability and refining these coordinate transformations.

### 4.2. Tilt Verification Implications

#### 4.2.1. Relationship Between Coil Tilt Angle and MEP Amplitude

**Figure 9:**
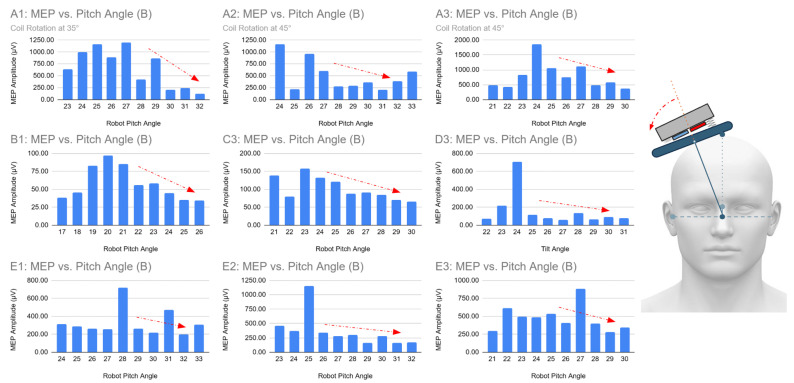
MEP vs. Pitch Angle Graphs showing similar patterns of MEP amplitude decreasing, visualized with dashed arrows, as coil is tilted downwards (higher robot pitch). The tilt direction that causes the pattern is also visualized with an arrow.

Across the 15 trials (five participants, three trials each), a clear trend emerged in 9 trials (60%) shown in Figure 9, where MEP amplitudes decreased progressively as the coil was tilted away from the top of the head (increasing robot pitch). In several trials, amplitudes remained high when the coil was tilted toward the top. Compared to the theoretical reference angle of 36° from the top of the head, these observations suggest that tilting away from the top reduces the coil’s alignment with the scalp surface, whereas tilting toward the top improves tangent orientation.

In the remaining six trials, the amplitude-to-tilt-angle relationship was less monotonic. However, peak responses still generally occurred near tilt angles that imply a tangent-to-scalp position. These deviations likely reflect inter-subject variability, such as head shape or hair thickness, or sensor noise.

#### 4.2.2. Sensor Ratio Correlation with MEP Amplitude

**Figure 10:**
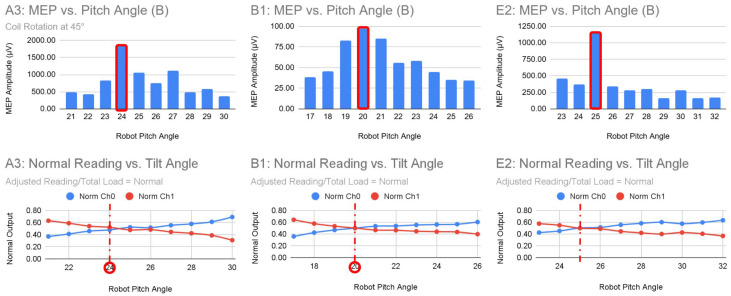
Peak MEP amplitudes, highlighted in red, in three representative trials align with dual sensor readings near a 1:1 ratio, indicating near-tangent coil–scalp orientation. Complete data for all trials are provided in Appendix A.

The dual textile sensors mounted vertically on the end-effector provided a reliable proxy for coil tilt. In 12 of 15 trials (80%) shown in Figure 10, the highest MEP amplitudes coincided with sensor readings approaching a 1:1 ratio, indicating symmetric displacement of the suspension system and, by extension, tangent-to-scalp orientation. This supports the principle that maintaining the coil tangent to the scalp optimizes stimulation.

Importantly, even in trials with noisy or non-intersecting sensor readings, the peaks in MEP amplitude still corresponded to conditions where the sensor ratio approached 1:1. This demonstrates that low-cost textile sensors can provide actionable real-time feedback on coil–scalp alignment, even without precise force sensors or complex impedance control, highlighting their practicality for robotic-assisted TMS.

#### 4.2.3. Inter-Subject Variability

MEP amplitude magnitudes varied substantially across participants, as expected due to differences in head shape and curvature, scalp thickness, individual MT levels, and coil-scalp contact geometry. However, despite these differences, the overall trends wherein the peak MEP amplitude occurs when near tangent-to-scalp alignment and reduced amplitude with increased tilt, remained consistently observable. This indicates that the textile sensor approach performs robustly across anatomical variability and does not require individual anatomical models.

### 4.3. Limitations in Sensor Consistency

While the textile sensors effectively detected tilt in most trials, some readings were ambiguous. Possible causes include slight sensor misalignment, inconsistent baseline calibration, or variability in head geometry. Additionally, only a single sensor reading was recorded per angle, increasing susceptibility to transient noise.

To improve reliability, future experiments will incorporate averaging over multiple samples (e.g., ≥10 readings per angle). This approach should reduce noise sensitivity and yield more stable indicators of tilt. Refinements to sensor mounting and calibration procedures are also expected to improve accuracy and repeatability.

### 4.4. Additional Findings

While not included in the main objectives of the experiment, a relationship between the MEP responses and total load readings was observed. Stimulations performed under moderate load ranges tended to produce stronger MEPs, while loads that were too low or too high resulted in weakened responses. Specifically, trials with a total load of 1500–4000 were associated with the highest and most consistent MEP amplitudes, whereas loads below 800 and above 6000 produced poor responses. These readings correspond to capacitance counts from the textile sensors, which are arbitrary units reflecting coil–scalp contact pressure rather than absolute force.

This suggests the presence of an optimal contact-pressure window for stimulation, consistent with studies [7] showing that both insufficient and excessive coil pressure can reduce scalp–coil coupling quality. This finding could be integrated into future closed-loop control strategies, allowing the robot to automatically approach the scalp and maintain optimal pressure during stimulation.

### 4.5. Future System Improvements

Short-term refinements include averaging multiple capacitance readings per angle, standardizing textile sensor calibration to reduce baseline drift, and upgrading camera calibration using an OpenCV checkerboard method to improve depth accuracy and facial landmark reliability. Additional improvements include refining pixel-to-point and point-to-robot transformations and incorporating coil thickness offsets into the kinematic model. Integration of the identified optimal load range into control logic could enable automatic maintenance or correction of coil pressure, advancing the system toward closed-loop operation. Furthermore, incorporating automated tilt correction to consistently achieve tangent-to-scalp orientation can further enhance positioning accuracy, although this feature has not yet been experimentally validated. Future studies could also compare setup time for C3 localization between the robotic-assisted system and traditional manual methods to demonstrate improved speed and usability. Conducting a comparison study between the accuracy and repeatability of this system and other similar systems would also help further verify the feasibility of the study.

Longer-term improvements, requiring additional resources, could significantly enhance system performance. A 6-DOF robot would allow full tilt compensation, while additional textile sensors or higher-resolution 3D/multi-view imaging could further improve scalp–coil coupling. Integration of neuroimaging, such as MRI-based targeting, would increase localization precision and allow individualized stimulation. Together, these upgrades outline a pathway toward a fully autonomous, high-precision robotic TMS positioning system.

## 5. Conclusions

This study demonstrates the feasibility of a robotic-assisted TMS positioning system integrating textile capacitive sensors and a 3D camera to support coil alignment and tilt detection. The C3 localization program reliably estimated and stimulated the C3 location with minimal calibration, while the dual textile sensors provided actionable, real-time feedback on coil tilting. Experimental results indicate that coil tilt and contact pressure significantly influence MEP amplitude, with optimal responses observed when the coil is in tangent to the scalp and within a moderate load range.

The proposed system shows promise for reducing operator dependency, improving reproducibility, and potentially reducing setup time compared to traditional manual localization methods. A tilt-correction algorithm utilizing differential sensor readings was implemented in the control software. However, its experimental validation was outside the scope of this study and is planned for future work. Short-term refinements, including improved sensor calibration, signal averaging, and camera calibration, are expected to further enhance system stability and estimation accuracy. Longer-term developments, such as the use of higher-DOF robotic platforms, additional sensing modalities, and integration of neuroimaging-based targeting, could advance the system toward more autonomous and higher-precision TMS delivery.

Because the proposed approach relies on scalp landmarks and does not incorporate individual neuroimaging data, anatomical targeting error relative to cortical anatomy or standardized spaces (e.g., MNI) was not quantified in this study. While the overall system includes a robotic arm and a commercial TMS device, the primary contribution of this work lies in reducing the cost and complexity of navigation and contact-sensing components. Compared to MRI-based neuronavigation or optical tracking systems, the proposed approach avoids external markers, dedicated tracking cameras, and force–torque sensors, thereby reducing system complexity and setup burden. Overall, these findings support the practical utility of combining low-cost sensing with robotic assistance for TMS applications and provide a clear roadmap for future enhancements aimed at improving precision, efficiency, and safety in both research and clinical settings.

## Figures and Tables

**Table 1:** Participant information and corresponding motor thresholds.

Subject	Dominant Hand	RMT (%)	120% RMT (%)
A	Right	62	74
B	Right	50	60
C	Right	55	66
D	Right	45	54
E	Right	64	77

**Table 2:** Summary of localization attempts and relevant notes.

Subject	Trial	Attempts	Remarks
A	1	5	Attempts 1–4: coil not positioned at 45 degrees
	2	1	Successful
	3	1	Successful
B	1	3	Recalibrated for height difference
	2	1	Successful
	3	1	Successful
C	1	4	Physically moved the cobot slightly backward
	2	2	Recalibrated target a bit closer to vertex
	3	1	Successful
D	1	2	Reverted to original target calibration
	2	1	Successful
	3	1	Successful
E	1	1	Successful
	2	1	Successful
	3	1	Successful

**Table 3:** Subject A Trial 3—Sample measurements.

Trial	Robot Pitch Angle	Total Load	Norm Ch0	Norm Ch1	MEP (µV)
Trial 3	21	1867	0.37	0.63	492.47
	22	1777	0.41	0.59	434.43
	23	1863	0.46	0.54	834.70
	24	1769	0.48	0.52	1861.40
	25	1669	0.52	0.48	1067.20
	26	1731	0.52	0.48	748.72
	27	1795	0.55	0.45	1108.10
	28	1752	0.58	0.42	483.87
	29	1741	0.61	0.39	572.38
	30	1614	0.69	0.31	367.76

**Figure A1:**
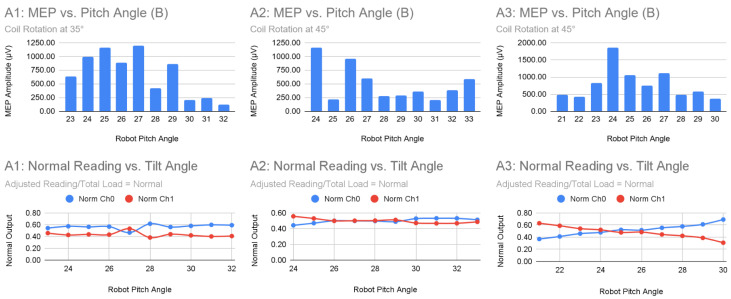
All trials from Subject A.

**Figure A2:**
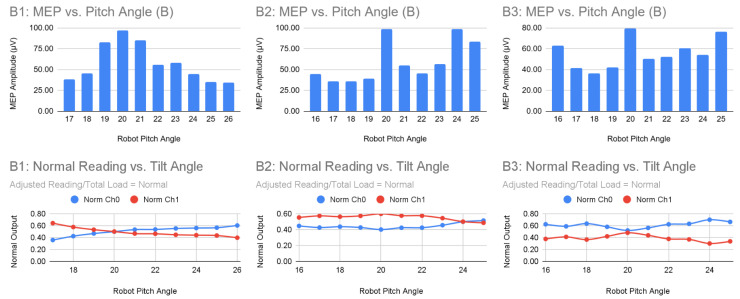
All trials from Subject B.

**Figure A3:**
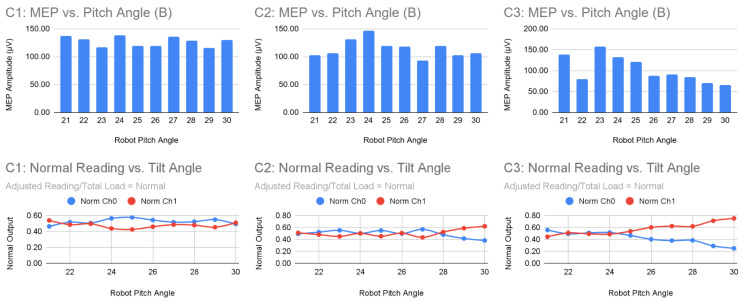
All trials from Subject C.

**Figure A4:**
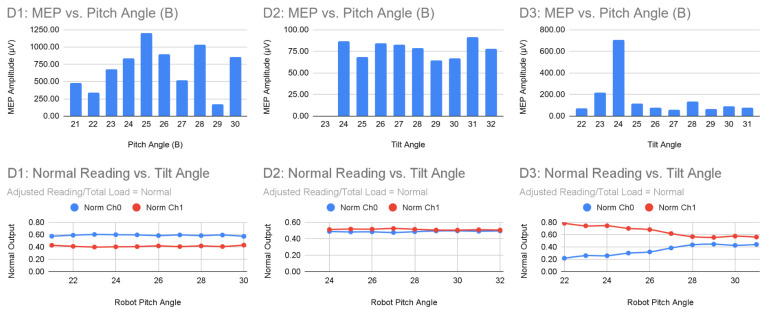
All trials from Subject D.

**Figure A5:**
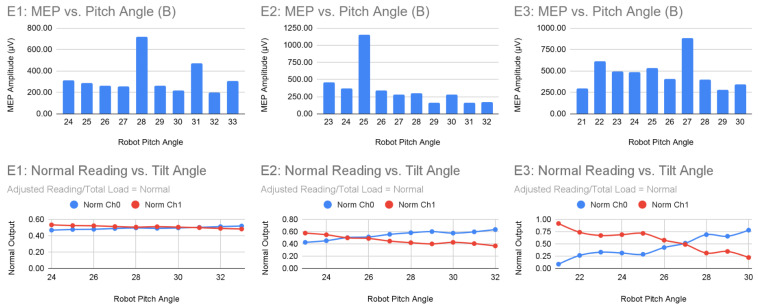
All trials from Subject E.

## Data Availability

The raw data supporting the conclusions of this article will be made available by the authors upon request.

## Abbreviations

The following abbreviations are used in this manuscript:

TMS	Transcranial Magnetic Stimulation
MDD	Major Depressive Disorder
PDD	Postpartum Depression
SUD	Substance Use Disorder
MRI	Magnetic Resonance Imaging
fMRI	Functional Magnetic Resonance Imaging
DOF	Degrees of Freedom
iRC	Igus Robot Control
CRI	Container Runtime Interface
MEP	Motor Evoked Potential
FDI	First Dorsal Interosseous
RMT	Resting Motor Threshold
MSO	Maximum Stimulator Output

## Appendix A

This appendix shows all recorded trials for the tilt verification experiment across all participants. Each figure includes the graphs for MEP amplitude vs. robot pitch angle, normalized textile sensor readings vs. robot pitch angle of all trials per subject. These graphs provide a complete overview of coil orientation effects on MEP amplitude and sensor symmetry, complementing the representative data presented in the main text.
